# A rapid real-time quantitative PCR assay to determine the minimal inhibitory extracellular concentration of antibiotics against an intracellular *Francisella tularensis* Live Vaccine Strain

**DOI:** 10.3389/fmicb.2015.01213

**Published:** 2015-11-03

**Authors:** Ronit Aloni-Grinstein, Ohad Shifman, Shlomi Lazar, Ida Steinberger-Levy, Sharon Maoz, Raphael Ber

**Affiliations:** ^1^Department of Biochemistry and Molecular Genetics, Israel Institute for Biological ResearchNess Ziona, Israel; ^2^Department of Pharmacology, Israel Institute for Biological ResearchNess Ziona, Israel

**Keywords:** *Francisella tularensis*, tularemia, antibiogram, intracellular infection, antibiotic susceptibility, qPCR, MIC, MIEC

## Abstract

*Francisella tularensis* is a highly virulent facultative intracellular bacterium. The lack of a safe and efficient vaccine makes antibiotics the preferred treatment. *F. tularensis* antibiotic susceptibility tests are based on the *in vitro* standard CLSI-approved microdilution method for determining the MIC. However, limited data are available regarding the minimal inhibitory extracellular concentration (MIEC) needed to eradicate intracellular bacteria. Here, we evaluated the MIEC values of various WHO-recommended antibiotics and compared the MIEC values to the established MICs. We describe a rapid 3-h quantitative PCR (qPCR) intracellular antibiogram assay, which yields comparable MIEC values to those obtained by the classical 72-h cfu assay. This rapid qPCR assay is highly advantageous in light of the slow growth rates of *F. tularensis*. Our results showed that the MIECs obtained for doxycycline, chloramphenicol and ciprofloxacin were indicative of intracellular activity. Gentamicin was not effective against intracellular bacteria for at least 32 h post treatment, raising the question of whether slow-penetrating gentamicin should be used for certain stages of the disease. We suggest that the qPCR intracellular antibiogram assay may be used to screen for potentially active antibiotics against intracellular *F. tularensis* as well as to detect strains with acquired resistance to recommended antibiotics.

## Introduction

*Francisella tularensis*, the etiological agent of tularemia, is a gram-negative facultative intracellular bacterium and is one of the most infectious pathogenic bacteria known (Dennis et al., 2001). As such, it is classified by the CDC as a category A agent.^1^ The route of infection and the bacterial subtype influence the disease severity. The most severe form of tularemia is caused by *F. tularensis* subsp. *tularensis* (type A), which is found in North America (Olsufjev and Meshcheryakova, 1983; Staples et al., 2006), while *F. tularensis* subsp. *holarctica* (type B) is responsible for tularemia across the entire northern hemisphere (Jusatz, 1961). The *F. tularensis* Live Vaccine Strain (LVS) (Eigelsbach and Downs, 1961; Tigertt, 1962) is an attenuated type B strain that does not cause disease in humans but that is virulent to mice and can infect various cell lines in culture, including human cell lines (Hall et al., 2007; Krocova et al., 2008). Indeed, many studies have documented the interaction of *F. tularensis* with phagocytic cell lines; however, recent studies have shown that *F. tularensis* can attach to and invade non-phagocytic cells such as lung epithelial cells, endothelial cells, fibroblasts and HepG2 cells (Qin and Mann, 2006; Hall et al., 2007; Moreau and Mann, 2013). Moreover, mice infected with *Francisella* LVS or SCHU S4 mutants, which do not efficiently replicate in macrophages, succumbed to infections, suggesting that the interaction of *F. tularensis* with non-phagocytic cells plays a role in its virulence strategy (Horzempa et al., 2010).

Due to the lack of an effective and safe vaccine, antibiotics are the central strategy for tularemia prophylaxis and treatment. According to the WHO guidelines (Anda et al., 2007), gentamicin and streptomycin (as an alternative) are the recommended antibiotics for treating hospitalized patients with severe tularemia. These aminoglycosides largely show *in vitro* bactericidal activity against *F. tularensis* types A and B and good clinical outcomes with minor relapses. The major drawbacks of aminoglycosides are their associated toxicity and the fact that they can be administered only parenterally (Enderlin et al., 1994; Boisset et al., 2014). Fluoroquinolones (ciprofloxacin and levofloxacin) and tetracyclines (doxycycline) are advocated as first-line antibiotics for mild to moderate tularemia cases (Limaye and Hooper, 1999; Perez-Castrillon et al., 2001; Ellis et al., 2002; Tarnvik and Chu, 2007). Ciprofloxacin, doxycycline and chloramphenicol are recommended for less severe cases or in a mass casualty setting such as post exposure prophylaxis (Dennis et al., 2001). Recently, it was shown that high-level fluoroquinolone-resistant mutants could be obtained easily and quickly (Sutera et al., 2014b). Moreover, some of the resistant mutants shared cross-resistance to other clinically relevant antibiotic classes, highlighting the notion that treatment should not rely only on organism identification but rather be based on the antimicrobial susceptibility testing (AST) results of the bacteria isolated from the patient. The conditions for performing AST of *F. tularensis* are defined by the CLSI guidelines (CLSI, 2010) and are based on the microdilution technique. This process involves the inoculation of a defined concentration of a bacterial suspension into a series of dilutions with progressively lower concentrations of the tested antibiotic. Growth is then monitored in defined growth medium and conditions. Due to the slow growth rate of *F. tularensis*, the AST assay requires 48 h before the Minimal Inhibitory Concentration (MIC) can be determined. The MIC is defined as the lowest antibiotic concentration that completely inhibits bacterial growth, as determined by the un-aided eye. Knowledge of the MICs allows the assignment of the bacteria to a susceptibility category (sensitive, intermediate, resistant or ‘non-sensitive’), which the clinician uses to evaluate the antibiotic treatment. Notably, however, this AST assay is conducted *in vitro* and thus does not address the antibiotic susceptibility within a living cell. As *F. tularensis* is a facultative intracellular bacterium, an antibiotic agent that will be effective against both the extracellular and intracellular forms is highly advantageous.

The goal of this study was to develop a rapid and simple assay to determine the effectiveness of antibiotics against the intracellular form of *F. tularensis*, as determined by calculating the Minimal Inhibitory Extracellular Concentration (MIEC) of the tested antibiotics. We have developed a rapid 3-h qPCR tool that provides comparable MIEC values to those obtained by the classical 72-h cfu assay. Our assay may help to determine the relationship between the pharmacokinetics (drug concentration at the site of infection) and the pharmacodynamics (drug effect on bacterial counts) in the complex environment of the *F. tularensis* intracellular infection. The experiments that were conducted at clinically relevant extracellular drug concentrations allowed for a comparison of the extracellular and intracellular activities of relevant antibiotics. Correlation between the MIEC and MIC values may predict a beneficial clinical outcome following treatment with the tested antibiotic.

## Materials and Methods

### Bacterial Strains, Media, and Growth Conditions

*Francisella tularensis* LVS (ATCC 29684) was grown on CHA plates (Difco 5.1% cystine heart agar, 1% hemoglobin) for 3 days at 37°C. For spinfection experiments, *F. tularensis* LVS was grown in TSBC broth (BD 3% tryptic soy broth, 0.1% L-cysteine) at 37°C under moderate agitation (150 rpm) to an optical density at 660 nm (OD_660_) of 0.15–0.3, representing the logarithmic phase.

### *F. tularensis* LVS Growth in Vero Cells

The Vero cell line (ATCC CCL-81) was grown in Dulbecco’s modified Eagle’s medium (DMEM) (Biological Industries, Beth Haemek, Israel) supplemented with 10% fetal calf serum, 2 mM glutamine and a 1:100 dilution of a non-essential amino-acid solution (Biological Industries, Beth Haemek, Israel). Cells were grown at 37°C in a 5% CO_2_ humidified incubator. For spinfection experiments (Peng et al., 2011; Bradburne et al., 2013), Vero cells were plated at a concentration of 2 × 10^5^ cells/ml in 24-well tissue culture plates (1 ml per well) and incubated for 24 h. Cells were infected with an *F. tularensis* LVS culture diluted in TSBC to OD_660_ = 0.15 (∼5 × 10^8^ cfu/ml) to yield a multiplicity of infection ratio of 1:3,000. The infected cells were centrifuged for 5 min at 600 *g* following a 55-min incubation period at 37°C in a 5% CO_2_ humidified incubator. To remove the extracellular bacteria, the cell monolayers were washed three times with sterile phosphate-buffered saline (PBS, Biological Industries, Beth Haemek, Israel) and then treated for 1 h at 37°C with 20 μg/ml gentamicin (Sigma G1397), followed by three washes with sterile PBS. The infection yield was determined by harvesting the cell fraction with 1 ml of 0.1% sodium deoxycholate (Sigma D6750) per well for 1 min, and cfu counts were determined by plating 10-fold dilutions on CHA plates. All experiments were run in duplicate, and the averages of the duplicate results are presented in the figures.

### MIC and MBC Determination

Antibiotic susceptibility tests were performed by the standard microdilution method, according to the CLSI guidelines for *F. tularensis* (CLSI, 2010). *F. tularensis* LVS was prepared by being plated on CHA plates and incubated for 3 days at 37°C in ambient air. Isolated colonies were suspended to OD_660_ = 0.075 (∼1–4 × 10^8^ cfu/ml) in cation-adjusted Mueller-Hinton broth (BBL, 212322) supplemented with 2% defined growth supplement (IsoVitaleX Enrichment; BBL 211876) and 3 μM hematin (Sigma 3281). The bacterial suspension was diluted 1:100 and inoculated in duplicate into 96-well microtiter plates containing twofold serial dilutions of each antimicrobial agent to yield approximately 5 × 10^5^ to 2 × 10^6^ cfu/ml in a final volume of 100 μl. The inoculum size was verified by plating 10-fold dilutions on CHA and incubating 3 days at 37°C for cfu counts. The microtiter plates were incubated in a plate reader (TECAN Sunrise or Infinite 200) for 48 h at 37°C in ambient air, and the absorbance at OD_630_ was read at 1-h intervals. The following antibiotics were used: doxycycline (Sigma D9891), ciprofloxacin (Ciproxin 200, Bayer), erythromycin (Sigma E5389), chloramphenicol (Sigma C0378) and gentamicin (Sigma G1397). The MICs of ciprofloxacin and doxycycline were determined by >10 independent repetitions, and those of gentamicin, chloramphenicol and erythromycin by four independent repetitions. The MIC was defined as the lowest concentration that reduced growth to less than 10% of the OD_630_ of the growth control, and the lack of visible growth was verified by the unaided eye. The Quality Control (QC) strains *Staphylococcus aureus* ATCC 29213 and *Escherichia coli* ATCC 25922 were used to ensure consistent MIC results within the defined reference MIC range (CLSI, 2015).

The minimal bactericidal concentration (MBC) were determined following the CLSI guidelines (CLSI, 1999). The MBC was determined by plating the content of the microtiter plate wells containing MIC and higher concentrations of antibiotics on CHA plates and incubating at 37°C for 5 days for viable cfu counts. The MBC was determined as the concentration that caused a 3-log decrease in cfu counts compared with the original inoculum. MBCs were determined in at least three independent repetitions.

### MIEC Determination

For intracellular antibiogram assays, spinfected cells were grown for 24 h in fresh supplemented DMEM containing 20 μg/ml gentamicin (Sigma G1397) and a twofold dilution series of the tested antibiotic. Post incubation, the medium from each well was collected to determine the cfu counts, and the cell monolayers were washed three times with sterile PBS, harvested by adding 1 ml of 0.1% sodium deoxycholate (Sigma D6750) to each well for 1 min and collected for both quantitative PCR (qPCR) analysis and bacterial cfu counts. The cell fraction and the medium were serially diluted and plated on CHA plates for cfu counts. The MIEC was determined as the minimal antibiotic concentration that prevents bacterial growth in the cellular fraction.

### Quantitative PCR Analysis

For qPCR analysis, a sample of 100 μl cell extract was first added to 100 μl Triton buffer (20% Triton X-100 (Sigma T8787) in TE buffer) and heated for 30 min at 100°C. DNA was then purified with the QIAamp DNA blood mini kit (QIAGEN 51104) and subjected to real-time PCR using the SensiFAST^TM^ Probe Lo-Rox kit (Bioline BIO-84005) with an Applied Biosystems 7500 real-time PCR system. DNA quantification was performed based on the gene *fopA*, as previously described (Versage et al., 2003). The primers (fopAF: ATCTAGCAGGTCAAGCAACAGGT, fopAR: GTCAACACTTGCTTGAACATTTCTAGATA) and the dual-labeled probe (fopAP: 6-FAM-CAAACTTAAGACCACCACCCACATCCCAA-BHQ-1) were ordered from Integrated DNA Technologies, Inc.

To determine the cfu/ml equivalents of the qPCR assay, 10-fold dilutions of *F. tularensis* LVS cultures, pre-quantified by the cfu counts, were used as a reference. Equivalent cfu/ml were determined from the standard curve using 7500 Real-Time PCR System Sequence Detection Software (v1.4, Applied Biosystems, Inc.).

### Immunolocalization of *F. tularensis* LVS in Cultured Vero Cells by Confocal Microscopy

Quantification of intra/extracellular bacteria using double-labeled immunofluorescence has been previously described (Law et al., 2014). Briefly, Vero cells were seeded on #1 glass cover slips in 24-well dishes and spinfected with *F. tularensis*. Cells were fixed with 4% paraformaldehyde (PFA, Gadot, Israel) for 10 min at 4°C, washed three times with PBS, and placed for 1 h in a blocking solution [10% normal goat serum (NGS) in PBS containing 0.05% Tween-20 (Sigma–Aldrich P5927)].

#### Staining of Extracellular Bacteria

The cells were incubated in a 1:500 dilution of primary rabbit anti-*F. tularensis* hyper-immune serum in an antibody cocktail solution (50% blocking solution/0.05% Tween-20/PBSX1) for 24 h at 4°C. Then, the slides were washed three times (5 min each) with washing buffer (1% blocking solution/0.05% Tween-20/PBSX1), incubated with secondary Alexa Fluor-488 (Molecular Probes, Life Technologies, Burlington, ON, Canada) at a 1:250 dilution in an antibody cocktail solution for 24 h at 4°C, and washed three times with washing buffer.

#### Staining of Intracellular Bacteria

Cell membranes were permeabilized [0.2% Triton X-100 (TX-100)/PBSX1] for 10 min, blocked again for 1 h [10% NGS in PBS containing 0.05% TX-100], incubated in a 1:500 dilution of primary rabbit anti-*F. tularensis* hyper-immune serum in antibody cocktail solution (50% blocking solution/0.05% TX-100/PBSX1) for 24 h at 4°C. Then, the membranes were washed three times (5 min each) with washing buffer (1% blocking solution/0.05% TX-100/PBS), and incubated with a mixture of secondary Alexa Fluor-594 (1:250 dilution) (Molecular Probes, Life Technologies, Burlington, ON, Canada) in an antibody cocktail solution for another 24 h at 4°C. In the last step, nuclei were stained with DAPI (1 μg/ml, Sigma D9542) for 5 min at room temperature. Following three additional washes, the cells were mounted on slides using Fluoromount G (Southern Biotech, Birmingham, AL, USA). Images were acquired using a Zeiss LSM710 confocal microscope (Zeiss, Oberkochen, Germany). From each slide, five random fields were scanned for fluorescence quantification. Analysis was conducted using Zen1 (Zeiss, Oberkochen, Germany). Fluorescence intensity parameters were statistically analyzed using a *t*-test followed by the Mann–Whitney *U* test. Uninfected cells and infected cells processed in the absence of anti *F. tularensis* hyper-immune serum served as negative controls.

## Results

### Invasion and Intracellular Growth of *F. tularensis* LVS in Vero Cells

**FIGURE 1:**
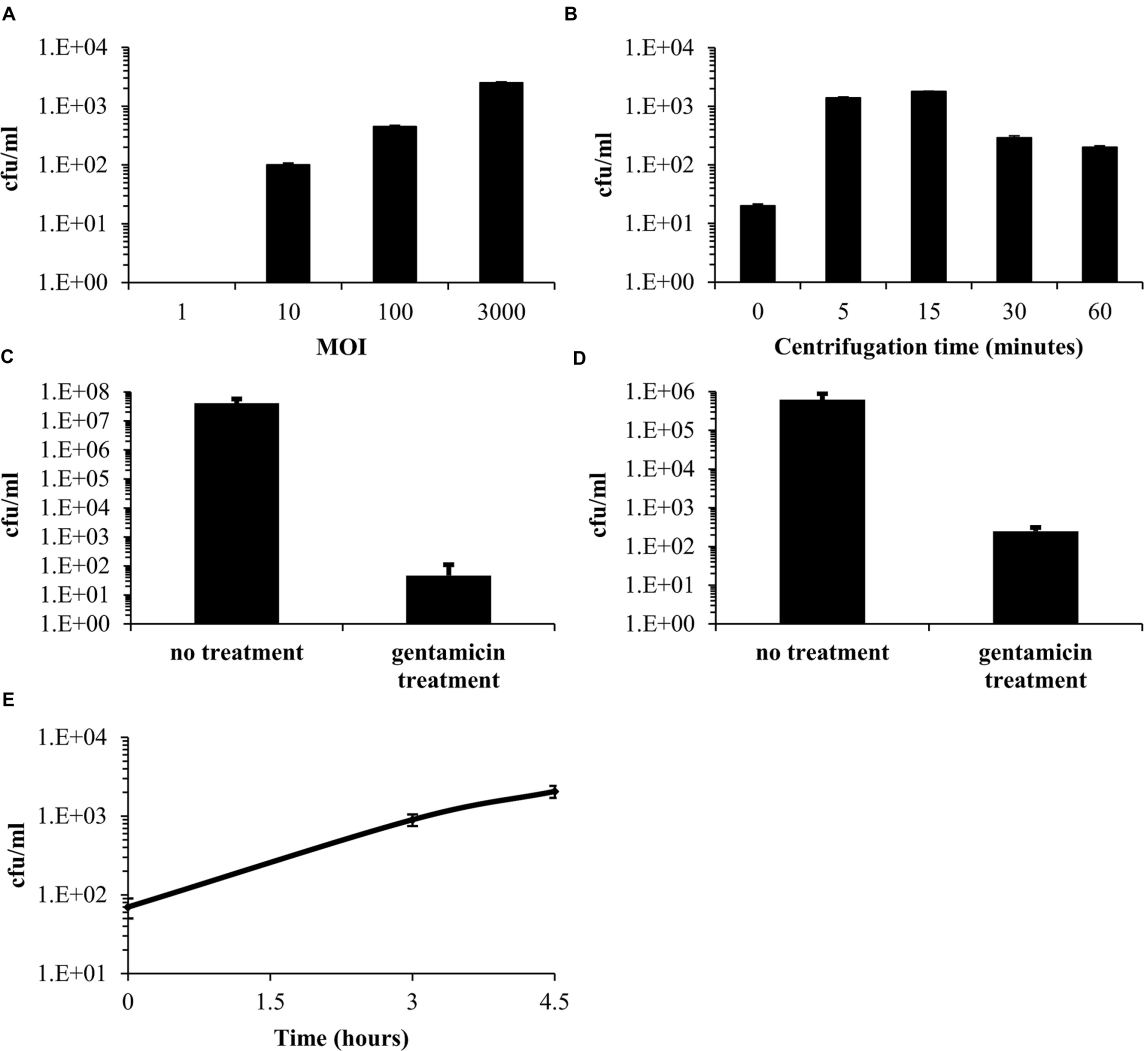
**Optimization of the infection procedure of Vero cells by *Francisella tularensis* LVS.** Vero cells were spinfected with *F. tularensis* LVS with a multiplicity of infection (MOI) ratio of 1, 10, 100, or 3,000. Bacteria within the cell fraction were extracted and quantitated using cfu counting **(A)**. The optimal centrifugation time was determined using an MOI of 1:3,000 by comparing the yields obtained without centrifugation to those obtained with various centrifugation times (5, 15, 30, and 60 min) **(B)**. The effect of a 1-h 20 μg/ml gentamicin treatment on the extracellular fraction **(C)**. The effect of a 1-h 20 μg/ml gentamicin treatment on the cellular fraction. **(D)** Concentrations of bacteria released from the cell fraction following infection and a 1-h gentamicin treatment **(E)**.

The entry of *F. tularensis* LVS into epithelial cells is believed to be less efficient than the uptake into macrophages (Hall et al., 2007; Bradburne et al., 2013); thus, we first optimized the infection procedure of *F. tularensis* LVS into Vero cells. We based our initial experiments on previous work describing the spinfection technique in Vero cells using centrifugation conditions of 600 *g* for 15 min at room temperature (Keren et al., 1995; Keysary et al., 1996). Using these conditions, followed by 45 min of incubation at 37°C in a 5% CO_2_ humidified incubator for a total infection period of 60 min, we found that a multiplicity of infection (MOI) of 1:3,000 yielded the maximal cell-associated bacteria from all tested MOIs (**Figure 1A**). We then worked to optimize the centrifugation time. Vero cells were spinfected with *F. tularensis* LVS at an MOI of 1:3,000 without centrifugation or with various centrifugation times (5, 15, 30, and 60 min). Following centrifugation, cells were incubated at 37°C in a 5% CO_2_ humidified incubator for a total infection period of 60 min. We found that both 5 and 15 min of centrifugation yielded the highest *F. tularensis* LVS cfu counts associated with the cell fraction. Cfu counts of 100s to several 1000s were consistently recovered from each well (**Figure 1B**). Following these results, all the experiments described in this work were performed using an MOI of 1:3,000 and 5 min of centrifugation following 55 min of incubation at 37°C in a 5% CO_2_ humidified incubator.

To remove the remaining free extracellular bacteria, supplemented DMEM medium containing 20 μg/ml gentamicin was added to the cell culture for 1 h. This resulted in a 4–5-log decrease of the cfu counts in the medium (**Figure 1C**). Surprisingly, although gentamicin does not accumulate in cells within an hour (Carryn et al., 2003), we also observed a 2–3-log decrease in viable bacteria counts in the cell fraction (**Figure 1D**), suggesting that most of the cell-associated bacteria (∼98%) were not located within the cells but were adhered to the cell surface, where they are partially exposed to the bactericidal effect of gentamicin. We then checked the cfu counts in the medium and found a continuous rise following infection (**Figure 1E**) and up to 48 h (data not shown). Of note, our supplemented DMEM control without Vero cells, supported growth of approximately 1 log/24 h of *F. tularensis* LVS (data not shown). This minor growth, which was not observed by others (Maurin et al., 2000; Ludo et al., 2008), cannot account for the majority of the bacteria that we found in the medium, suggesting a continuous release of the adhered bacteria to the external medium. The presence of bacteria in the medium indicated a need for the continuous presence of gentamicin in the MIEC determination assay to prevent extracellular growth and the possible reinoculation of cells by these bacteria.

**FIGURE 2:**
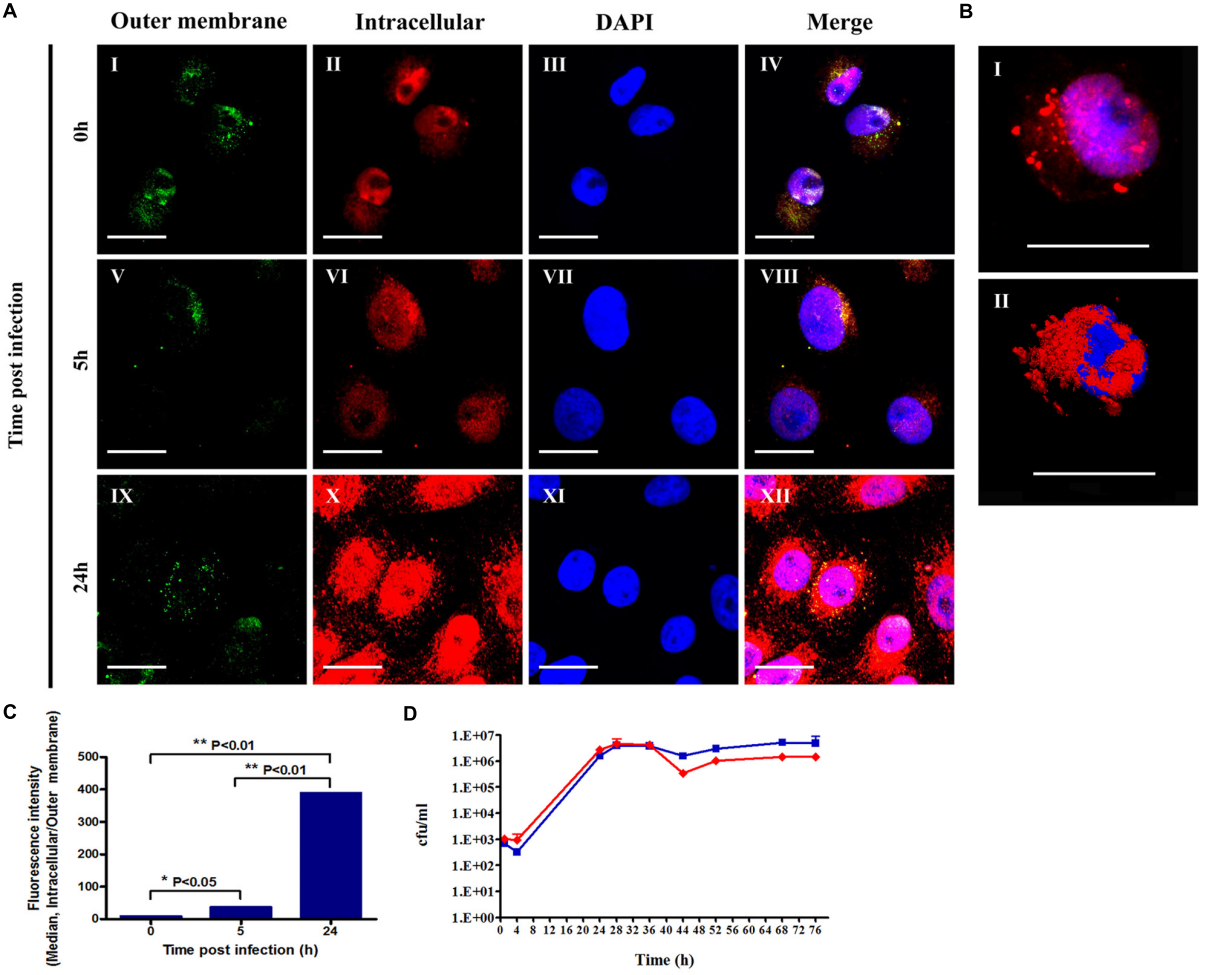
**Assessment of the intracellular and outer membrane-associated *F. tularensis* bacteria.** Vero cells infected with *F. tularensis* LVS bacteria were analyzed using a confocal microscope. All cell cultures were treated for 1 h with 20 μg/ml gentamicin following 3xPBS washes. Cell cultures of 5 and 24 h were grown in medium containing 20 μg/ml gentamicin. Double-labeled immunofluorescent images of *F. tularensis* LVS infections of Vero cells after 0, 5, and 24 h are presented in **(A)**. Bacteria bound to the outer membrane appear green **(AI,V,IX)** or yellow in merged panels **(AIV,VIII,XII)**, while intracellular bacteria appear exclusively red **(AII,VI,X)**. Nuclei stained with DAPI are presented in images **(AIII,VII,XI)**. The frame size in **(AI–XII)** is 512 × 512, the dimensions are 70.71 × 70.71 μm, the magnification is x120, and the scale bar = 20 μm. A representative optical slice of the 3D analysis of a nucleus surrounded by bacteria in infected cells is presented in **(B)**. The images represent fluorescent **(BI)** and surface projections **(BII)** of the same cell 5 h post infection. Frame size: 512 × 512; dimensions: 73.15 × 73.15 × 23 μm; magnification: x120; scale bar = 20 μm. The ratio between the fluorescent intensity of intracellular versus outer membrane-associated bacteria labeling is presented in **(C)**. All experiments were performed in duplicate and repeated three times. Bacterial growth curves in the cellular fraction of Vero cells were monitored by cfu counts in the presence (red line) or absence of gentamicin (blue line). The experiment was performed in duplicate **(D)**.

To verify that *F. tularensis* LVS indeed invaded Vero cells and was not merely associated with the cell fraction, we used confocal microscopy to ascertain the bacterial localization within the cell fraction. Using differential staining conditions, we could discriminate the outer membrane-associated bacteria (**Figure 2AI**) from the intracellular *F. tularensis* LVS (**Figure 2AII**), and we thus confirmed the presence of the bacteria within the Vero cells (**Figure 2AIV**). No florescence intensity was observed in non-infected cells and in the absence of anti-*F. tularensis* serum (data not shown).

The low invasion rates of *F. tularensis* LVS in Vero cells compared with those reported for macrophage cell lines (Hall et al., 2007) may raise the question of whether *F. tularensis* is capable of replicating in Vero cells. To clarify this question we measured the fluorescence intensity of both intracellular and outer membrane-associated bacteria. Nuclei stained with DAPI are presented in **Figures 2AIII,VII,XI** and merged images of DAPI, outer membrane and intracellular bacteria labeling are demonstrated in **Figures 2AIV,VIII,XII**. We found that at 5 and 24 h post infection there was a significant increase in the fluorescence intensity of intracellular bacteria over time, as shown in **Figures 2AII,VI,X**. The bacteria were observed within the cytosolic compartment surrounding the nucleus (**Figure 2B**). The fluorescence intensity of the outer membrane-associated bacteria was similar immediately and 5 h post infection but decreased by threefold at 24 h post infection (**Figures 2AI,V,IX**). We postulate that the fluorescence of the outer membrane-associated bacteria represents bacteria that are embedded in the Vero cell membranes as the result of the centrifugation process. The ratio of intracellular fluorescence to outer membrane fluorescence increased dramatically within 24 h (**Figure 2C**), suggesting that the invading bacteria indeed replicated within the Vero cells.

To quantify the growth of the intracellular bacteria, we monitored the intracellular growth rates of the bacteria following infection. To verify that the scored bacteria were intracellular, cultures were grown in parallel with and without the addition of 20 μg/ml gentamicin to the cell growth medium. We found that despite the low invasion rates, *F. tularensis* LVS was capable of replicating in Vero cells, as shown in **Figure 2D**. The bacterial load increased by ∼1,000-fold in 24 h, reaching the late exponential phase. A similar rate of growth was documented by others in optimized modified Mueller-Hinton broth, in macrophages, HEp-2 cells, human bronchial epithelial cells and A549 tissue culture cells (Lindemann et al., 2007). The addition of gentamicin did not impair the growth of intracellular bacteria for at least 32 h, suggesting that the observed increase in bacterial counts was of an intracellular origin. These results are in agreement with previously published data demonstrating that the activity of aminoglycosides against intracellular *F. tularensis* increases progressively with time and reaches significant intracellular levels only after 48 h (Tulkens and Trouet, 1978; Maurin and Raoult, 1993).

### The Determination of MIEC Values in Epithelial Vero Cells

**FIGURE 3:**
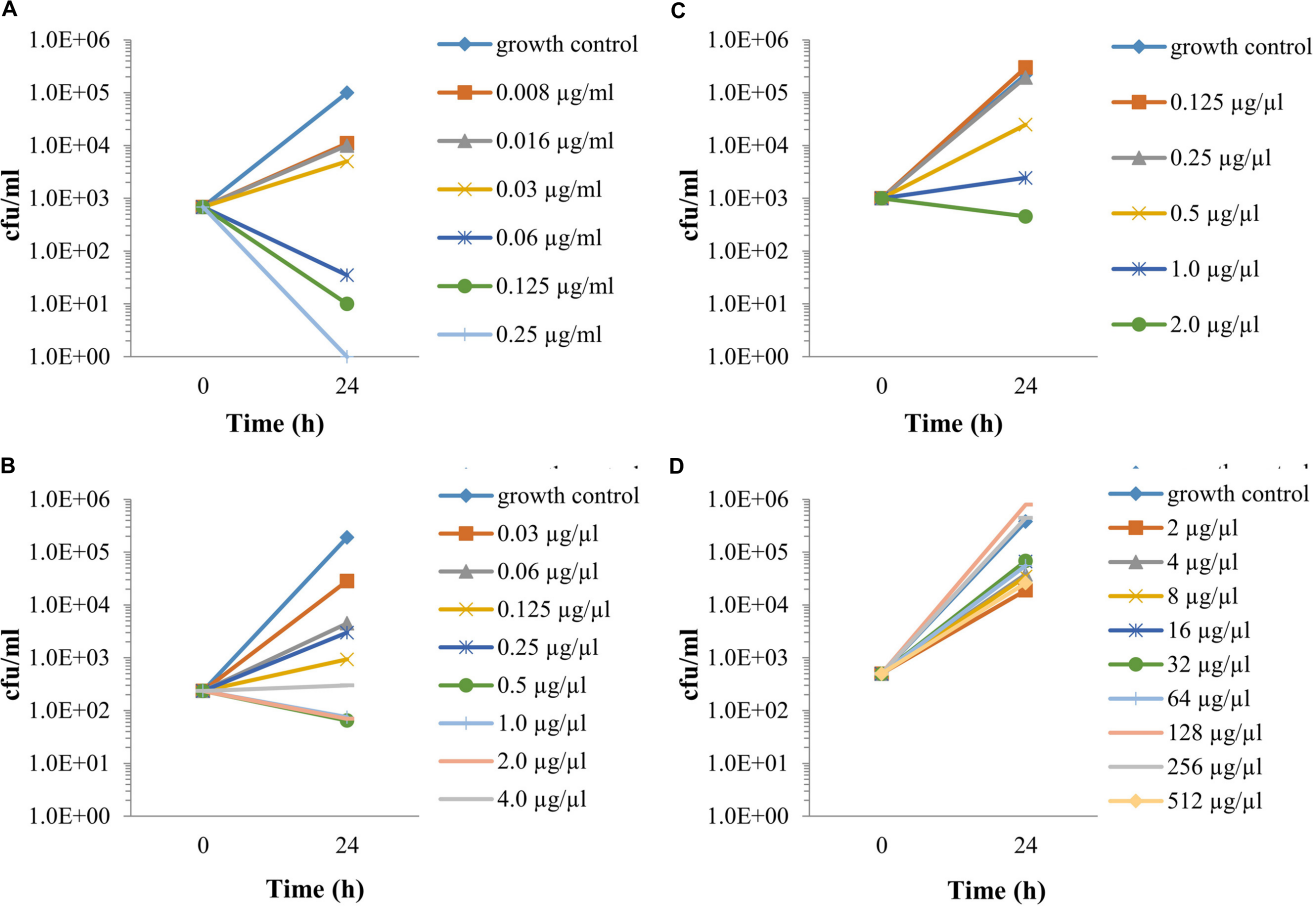
**The intracellular activities of antibiotics, as determined by cfu counting.** Vero cells were spinfected (MOI 1:3,000) with *F. tularensis* LVS, treated for 1 h with 20 μg/ml gentamicin, following 3xPBS washes. Fresh supplemented DMEM containing 20 μg/ml gentamicin and the tested antibiotic at a twofold dilution was added. The plates were incubated at 37°C in 5% CO_2_ atmosphere. After 24 h, the cell fractions were harvested and serially diluted by 10-fold in PBS following plating on CHA plates and were incubated for 72 h for cfu counts. MIECs were defined as no growth based on the initial infected concentration. Ciprofloxacin **(A)**, doxycycline **(B)**, chloramphenicol **(C)** and erythromycin **(D)**. All experiment were performed in duplicate. The number of repetitions for each antibiotic is stated in **Table 1**. The graphs are representatives of the repetitions.

**FIGURE 4:**
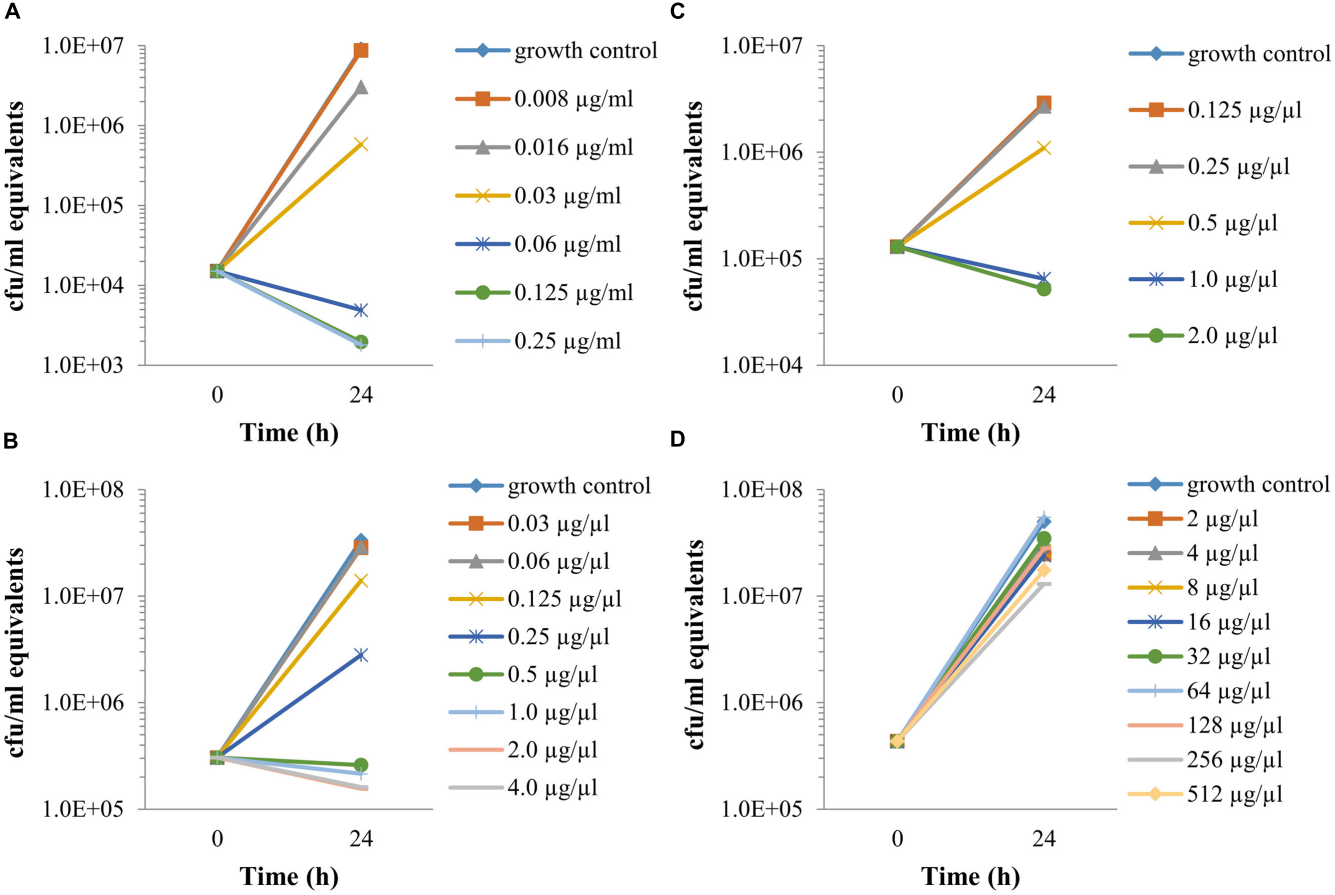
**The intracellular activities of antibiotics, as determined by qPCR.** Vero cells were spinfected (MOI 1:3,000) with *F. tularensis* LVS, treated for 1 h with 20 μg/ml gentamicin following 3 PBS washes. Fresh supplemented DMEM containing 20 μg/ml gentamicin and the tested antibiotic at a twofold dilution was added. After 24 h, the cell fractions were harvested, and a sample of 100 μl cell extract was added to 100 μl Triton buffer and heated for 30 min at 100°C. DNA was then purified and subjected to qPCR to determine PCR-equivalent cfu values. MIECs were defined as no growth based on the initial infected concentration. Ciprofloxacin **(A)**, doxycycline **(B)**, chloramphenicol **(C),** and erythromycin **(D)**. All experiment were performed in duplicate. The numbers of repetitions for each antibiotic is stated in **Table 1**. The graphs are representatives of the repetitions.

The exposure to different antibiotic concentrations was initiated 1 h after the infection of Vero cells with *F. tularensis* LVS, and the MIECs were determined 24 h following the initiation of antibiotic treatment, during the late logarithmic phase (**Figure 2D**), both by the cfu method following 3 days of incubation (**Figure 3**) and by the 3-h qPCR method (**Figure 4**). The antibiotic agents ciprofloxacin, doxycycline and chloramphenicol were selected because they are recommended for treatment in a contained casualty setting (Dennis et al., 2001), although the use of chloramphenicol is limited to exceptional cases due to the serious side effects associated with the drug (Kreizinger et al., 2013). *F. tularensis* LVS was shown to be resistant to azithromycin (Ahmad et al., 2010) and to erythromycin (Biswas et al., 2008). Resistance to erythromycin was shown to correlate with a point mutation in domain V of the 23S rRNA, which could explain the heterogeneity in susceptibility to erythromycin for *F. tularensis* strains (Biswas et al., 2008). Erythromycin was chosen to show the validity of the qPCR assay in an extreme case of resistance to a tested antibiotic. The MIECs determined by the cfu method (**Figure 3**) and qPCR method (**Figure 4**) were within one dilution of each other, despite the different readouts obtained by the two methods. The differences can be attributed to the methods of measurement. While the cfu counts assay measures only viable bacteria, the qPCR technique measures also non-culturable forms or killed bacteria that are in the process of DNA degradation. MIEC values were presented as a range when different values were obtained in repetitions (**Table 1**). The MIECs of ciprofloxacin, doxycycline and gentamicin were determined by five repetitions, of chloramphenicol by two repetitions and of erythromycin by one repetition.

**Table 1:** The activities of antibiotics against *Francisella tularensis* Live Vaccine Strain (LVS), as determined *in vitro* by the broth microdilution method (MIC) vs. the intracellular activity determined by cfu and qPCR (MIEC).

Antibiotic agent	^∗^MIC (μg/ml)	^∗∗^MIEC (μg/ml)	
		
		by CFU	by qPCR
Ciprofloxacin	0.008–0.016	0.06–0.25	0.06–0.25
Doxycycline	0.06–0.25	0.125–0.5	0.125–0.5
Chloramphenicol	0.5–1	1.0–2.0	1.0–2.0
Gentamicin	0.03–0.06	>20	>20
Erythromycin	>256	>256	>256


*^∗^MIC-minimal inhibitory concentration. ^∗∗^MIEC-minimal inhibitory extracellular concentration. MICs were determined for ciprofloxacin and doxycycline by 10 repetitions and for gentamicin, chloramphenicol and erythromycin by four repetitions. MIECs were determined for ciprofloxacin, doxycycline and gentamicin by five repetitions, for chloramphenicol by two repetitions and for erythromycin by one repetition. All experiments were performed in duplicate.*

### Standard MIC and MBC Determination

The MIC value was defined as the lowest concentration that reduced growth to less than 10% of the OD_630_ of the no-antibiotic growth control (equivalent to no visible growth by the aided eye). MIC values are presented in **Table 1** as a range to include all the values obtained in repetitions. *F. tularensis* LVS displayed the expected ‘susceptible’ category, as determined by MICs of 0.008–0.016 μg/ml, which is ≤0.5 μg/ml for ciprofloxacin, 0.06–0.25 μg/ml, which is ≤4 μg/ml for doxycycline, 0.03–0.06 μg/ml, which is ≤4 μg/ml for gentamicin, and 0.5–1 μg/ml, which is ≤8 μg/ml for chloramphenicol. As expected (Keim et al., 2007; Biswas et al., 2008), the *F. tularensis* LVS showed a high MIC value (>256 μg/ml) to erythromycin (**Table 1**), indicating the resistance of LVS. Notably, however, there are no susceptibility categories of macrolides for *F. tularensis*, as it is not a recommended compound for clinical treatment. The MBC values, which represent the survival of the bacteria during the 48 h of the AST in growth inhibitory concentrations (i.e., the MIC and higher concentrations), were determined as 99.9% reduction in cfu counts compared with the initial inocula. An MBC/MIC ratio <8 was considered bactericidal, and a ratio ≥8 was considered as a bacteriostatic mode of action (Pankey and Sabath, 2004). As expected, doxycycline showed a bacteriostatic mode of action, as noted by an MBC value of 32 μg/ml, and an MBC/MIC ratio of ≥64. The expected bactericidal mode of action is indicated for ciprofloxacin, as MBC values were in the range of 0.032–0.064 μg/ml and an MBC/MIC of ≤4. Gentamicin also demonstrated the expected bactericidal mode of action, with an MBC/MIC ratio of 1–2. Chloramphenicol, which is known to exhibit both bacteriostatic and bactericidal actions, depending on the bacteria, showed a clear bactericidal mode of action against *F. tularensis* LVS, with an MBC of 4 μg/ml and an MBC/MIC ratio of 4. Thus, as far as the *in vitro* activity indicates, all four recommended antibiotics are expected to be efficacious against the extracellular form of the bacteria under conventional treatment.

## Discussion

Tularemia is considered a re-emerging disease. In the last decade, sporadic cases and outbreaks in various locations in the U.S. and Europe have been documented (CDC, 2013; Johansson et al., 2014; Mailles and Vaillant, 2014). Moreover, *F. tularensis* is defined as a category A potential bioterrorism agent by the CDC.^2^ The lack of an effective vaccine places antibiotics as the only treatment option. In addition to naturally occurring antibiotic resistance such as the resistance of *F. tularensis* subsp. *holarctica* biovar II to erythromycin in contrast to the erythromycin-susceptible biovar I (Keim et al., 2007), *in vitro* selected resistance (Gestin et al., 2010; Sutera et al., 2014b) may also be of concern if misused for bioterrorism. To develop new or alternative antibiotic treatments, determining whether both the extracellular and intracellular forms of the bacteria are sensitive to the tested antibiotic is important. Moreover, due to different sub-cellular pharmacokinetics and variables such as specific influx/eﬄux ratios, binding, tissue metabolism, and the accumulation and bioavailability of an active antibiotic form, no simple correlation can predict a connection between the cellular concentration of antibiotics and the bacterial responsiveness (Van-Bambeke et al., 2006). Compared to the MIC AST, the MIEC test adds another level of prediction, as the assay reflects the dynamic effects of drugs on the host cell-intracellular microorganism relationship. Unlike animal models, which cannot be standardized or compete with the *in vitro* assay time scales, the cell line model has the advantage of both the ‘static’ antibiotic concentration of the *in vitro* microdilution method, and some aspects of the *in vivo* host-pathogen interaction. These include differences in pharmacokinetics at various internal compartments and pharmacodynamic properties such as local microenvironment pH differences, binding of the antibiotics to internal structures/proteins and interaction with specific degrading/modifying enzymes. Yet, the cell line model has its drawbacks and cannot replace the final *in vivo* challenge in the animal model, as factors like C_max_, serum elimination half-life rates, serum protein binding, involvement of the immune system and other host defense mechanisms, cannot be evaluated. Nevertheless, screening for new antibiotic compounds against intracellular bacteria in the MIEC assay may be used to predict antimicrobial efficiency, making the MIEC assay a relevant approach that can reduce the use of animals for testing non-relevant compounds.

Usually, the intracellular activity of a tested antibiotic against intracellular facultative microorganisms such as *F. tularensis* is measured by the viable bacterial counts, as determined by measuring the cfu. Because *F. tularensis* is a slow-growing bacterium, this assay is time consuming, and as many as 3 days may be needed to obtain 1–2 mm colonies. Thus, we have adapted a qPCR assay that quantifies the inhibition of bacterial growth within 3 h. We compared the MIECs obtained by the qPCR and cfu assays and examined four of the first-line recommended antibiotics for the treatment of tularemia (ciprofloxacin, doxycycline, gentamicin and chloramphenicol). We chose these antibiotics since they have standard test conditions and susceptibility categories for *F. tularensis* by the clinical and laboratory standard institute (CLSI, 2010). Furthermore, they represent different modes of action, usually aminoglycosides and fluoroquinolones being bactericidal (concentration dependent mode of killing), tetracyclines considered bacteriostatic (exposure time-dependent killing) and the nature of chloramphenicol mode of action is dependent on the bacteria (bactericidal for *F. tularensis*). In addition, we chose erythromycin, which is effective against several subtypes of *F. tularensis* but not against *F. tularensis* LVS, to show the validity of the qPCR-MIEC assay also with resistant strains. Our results show that the MIECs obtained by cfu counts were the same as those obtained by qPCR, suggesting that during the evaluation of a virulent strain, qPCR may serve as a quicker alternative for determining the MIECs. Moreover, the MIECs obtained for doxycycline and chloramphenicol were similar to the *in vitro* MIC values, indicating that both antibiotics were effective against the intracellular as well as the extracellular bacteria at the time point tested. These results are in agreement with the fact that the accumulation level at equilibrium, that is, the ratio between the cellular concentration and the extracellular concentration, of tetracyclines in cultured macrophages is 1–4 and that the time from influx to equilibrium is only minutes (Carryn et al., 2003; Van-Bambeke et al., 2006). However, the MIECs of ciprofloxacin were 8- to 16-fold higher than the MICs, suggesting that the intracellular bacteria experienced some degree of protection. Whether the internal ciprofloxacin concentration, which was shown to be 4–7 times the extracellular concentration in neutrophils is fully active is not clear (Easmon and Crane, 1985). This antibiotic may be subjected to strong eﬄux activity from the phagosomes in which intracellular *F. tularensis* resides or may be partly inactive, as ciprofloxacin has only 1/15 of the activity against internal bacteria as it does against extracellular bacteria, despite similar internal concentrations in macrophages (Carryn et al., 2002). The MIEC values for erythromycin and gentamicin were high. The high erythromycin MIEC correlated with the high MIC due to the intrinsic resistance of this strain of *F. tularensis* to erythromycin. However, the MIC for gentamicin was low (0.03–0.06 μg/ml), and the MIEC was high (>20 μg/ml), suggesting that *F. tularensis* LVS is sensitive to gentamicin but that the intracellular concentration of gentamicin 24 h post treatment is not sufficient to achieve growth arrest (**Figure 2D**). Indeed, the time needed for gentamicin to reach equilibrium in several cell types is several days (Van-Bambeke et al., 2006). Our results show that only after 2 days of gentamicin treatment, an effect on the growth of intracellular bacteria was observed. Others report that gentamicin given 24 h post infection to SCHU S4-infected mice is expected to start accumulating in phagocytic vesicles only several days after treatment initiation, and the effect observed in the infected mice is likely due to the suppression of replication of the extracellular bacteria, leading to decreased dissemination of bacteria from the lungs to distal organs (Sutherland et al., 2012). Notably, the retrospective report on treatment failure in tularemia patients who did not receive prompt treatment (Kaya et al., 2012) showed that treatment failed in 4 out of 7 patients treated with gentamicin but in only one patient out of 13 receiving streptomycin and in one patient out of seven receiving ciprofloxacin, emphasizing the differences in responsiveness to gentamicin during different phases of the disease.

Recently, a new dye uptake assay that tests the activity of antibiotics against intracellular *F. tularensis* was described (Sutera et al., 2014a). This assay takes advantage of the cytotoxic effect of bacteria on eukaryotic cells. The activity of the tested antibiotic is scored by its potential to prevent cytotoxic effects by inhibiting bacterial activity. Because this assay does not directly measure the effect of the tested antibiotic on the bacteria but instead assesses a secondary effect, the assay is time consuming, taking 5 days. Notably, the direct cfu assay is also on the scale of 3 days. In contrast, our qPCR assay directly measures the effect of the antibiotic on the bacteria, and the results are obtained within 1 day following the initiation of treatment. Thus, the qPCR assay is superior to the dye uptake and cfu assays in terms of time efficiency.

Although the study by Sutera et al. (2014a) and ours examined different strains of *F. tularensis* subsp. *holarctica*, the MICs for ciprofloxacin, doxycycline and gentamicin were comparable. However, while the MIECs for ciprofloxacin and doxycycline were also comparable, the values for gentamicin were very different. Whereas the MIEC was above 20 μg/ml in our study, the Sutera et al. (2014a) study found an MIEC of 2 μg/ml. These differences may be attributed to the different durations of the different assays. In the qPCR assay, the bacteria are scored for MIEC determination within 24 h of the initiation of treatment. During this period, gentamicin does not usually penetrate the cells; thus, high MIECs are expected. However, gentamicin does penetrate the cells during the 5-day incubation period of the dye uptake assay. Indeed, the MIECs were lower (2 μg/ml) than those we observed but were somewhat higher than the MIC values, indicating that gentamicin is less effective against intracellular *F. tularensis*, as previously suggested by the authors (Sutera et al., 2014a). The discrepancy in the MIEC values obtained by the different assays may be of clinical relevance both in the evaluation of new antibiotics and in determining effective treatment options for different stages of the disease. In cases in which an immediate effective response against the intracellular bacteria is required, gentamicin or other slow-penetrating antibiotics may not be the preferred choice.

## Conclusion

We showed that *F. tularensis* LVS bacteria are capable of invading and replicating in epithelial Vero cells. The exponential phase lasted for 24 h and allowed a 1,000-fold increase in the initial load. The bacteria were observed in the cytosolic compartment surrounding the nucleus. Indeed, *F. tularensis* was shown by others to develop mechanisms of adaptation to cytosolic proliferation (Celli and Zahrt, 2013). We used this model to study the effectiveness of various first-line antibiotics against the intracellular bacteria and found that the qPCR assay provided accurate MIEC values compared to those obtained by cfu counts. Furthermore, the qPCR assay is quicker than the cfu assay. This approach is of great value in light of the slow growth rates of *F. tularensis*. Thus, the qPCR intracellular antibiogram assay may facilitate the future standardization of intracellular antibiogram processes and may help to evaluate the effectiveness of new compounds against the intracellular form of *F. tularensis*. Moreover, high MIEC values indicate a low intracellular activity/concentration and may explain treatment failure.

## Author Contributions

Research project design: RA-G, OS, SL, IS-L, and RB. Experiments: RA-G, OS, SL, SM, and RB. Writing: RA-G and RB.

## Conflict of Interest Statement

The authors declare that the research was conducted in the absence of any commercial or financial relationships that could be construed as a potential conflict of interest.

## Footnotes

1 http://www.bt.cdc.gov/agent/agentlist.asp

2 http://www.bt.cdc.gov/agent/agentlist.asp
